# Alteration of specific cytokine expression patterns in patients with breast cancer

**DOI:** 10.1038/s41598-019-39476-9

**Published:** 2019-02-27

**Authors:** Kosuke Kawaguchi, Masashi Sakurai, Yasuko Yamamoto, Eiji Suzuki, Moe Tsuda, Tatsuki R. Kataoka, Masahiro Hirata, Mariko Nishie, Takashi Nojiri, Motofumi Kumazoe, Kuniaki Saito, Masakazu Toi

**Affiliations:** 1 Department of Breast Surgery, Kyoto University Graduate School of Medicine, Kyoto, Japan; 2 Human Health Sciences, Graduate School of Medicine and Faculty of Medicine, Kyoto University, Kyoto, Japan; 3 Department of Disease Control and Prevention, Fujita Health University Graduate School of Health Sciences, Toyoake, Aichi Japan; 4 Department of Diagnostic Pathology, Kyoto University Hospital, Kyoto, Japan; 5 Department of General Thoracic Surgery, Higashiosaka City Medical Center, Osaka, Japan; 6 Department of Bioscience and Biotechnology, Faculty of Agriculture, Kyushu University, Fukuoka, Japan

## Abstract

Systemic inflammation has been associated with aggressive tumor growth, invasion, and metastasis. Here we performed a comprehensive analysis of 26 kinds of inflammatory cytokine expression patterns among 185 patients with breast cancer and 54 healthy volunteers followed by chemometric analysis. We identified the specific cytokine expression patterns of breast cancer patients compared to healthy volunteers with (1) VEGF, IL-9, GM-CSF, IL-13, IL-4, and IFNγ, (2) IL-8, IL-10, IL-12, IL-5, IL-7, IL-1α, GCSF, IL-1β, and TNFα and (3) IL-2, Eotaxin, MIP1β, MIP1α, IL-17, and bFGF. Among the patients with breast cancer, we identified the specific cytokine signature of metastatic patients compared to non-metastatic patients. We also established a mathematical model for distinguishing patients with breast cancer from healthy volunteers and metastatic patients from non-metastatic patients. This cytokine network analysis could provide new insights into early intervention and effective therapeutic strategy for patients with breast cancer.

## Introduction

Inflammation plays a crucial role in the immune system and defense against several pathogens, including viruses and bacteria, and has been proposed to mediate the initiation and promotion of tumors, angiogenesis and metastasis^1^. Inflammatory cells are attracted by oncogenic changes, hypoxia, cytokines, and chemokines, among other factors. Inflammation of a tumor microenvironment comprises infiltrating immune cells and activated fibroblasts, which secrete cytokines, chemokines, and growth factors to which the tumor responds^2^. Moreover, a recent study has shown that inflammation signaling, especially involving IL-6/JAK2/Stat3, plays a crucial role in the stem cell-like characteristics of breast cancer (BC)^3^.

Measurement of cytokines in serum involves well-established methods in clinical practice. IL-6 may stimulate cancer cell growth and contribute to recurrence and metastasis in BC^4^, with GCSF, IL-6, and IL-17 able to distinguish between BC and no-cancer groups^5^. While most studies have found elevated levels of cytokines in patients with BC, a study of 90 patients with BC and 15 healthy volunteers based on univariate analysis found no differences in baseline cytokine levels between the cancer patients and the healthy volunteers, as evidenced by plasma levels of IL-1β, IL-6, IL-8, IL-10, IL-12 and TNF-α^6^. However, the direct correlation between inflammatory cytokine levels and clinical outcomes has not been evident in patients with BC.

Chemometrics-driven evaluations, especially principal component analysis (PCA), are among the data analysis methods used for statistical inference of low-dimensional parameters with high-dimensional data^7^; furthermore, many valuable diagnostic data have been revealed by PCA, in particular, in comprehensive gene analysis^8^. Recently, we reported that mRNA expressions of PD-L1, FOXP3, CD80, CD40 and CD14 in peripheral blood mononuclear cells are affected by disease development and progression^9^. These data revealed that circulating immune cells were affected by BC development and progression.

Here we performed a comprehensive analysis of inflammatory cytokine in serum derived from 185 patients with BC and 54 healthy volunteers. Our study showed the patients with BC had three different cytokine expression patterns: (1) VEGF, IL-9, GM-CSF, IL-13, IL-4, and IFNγ; (2) IL-8, IL-10, IL-12, IL-5, IL-7, IL-1α, GCSF, IL-1β, and TNFα; (3) IL-2, eotaxin, MIP1β, MIP1α, IL-17, and bFGF. Our data showed that BC patients were distinguishable from healthy volunteers. In the patients with BC, metastatic human epidermal growth factor receptor (HER2)-positive BC patients and triple-negative BC (TNBC) patients showed the specific signature plotting pattern. Furthermore, these cytokine profiles correlated with clinical outcomes in BC.

## Results

### Patient characteristics

Data on 185 BC patients and 54 women healthy volunteers were available. All of the samples were collected at the time of diagnosis. One-quarter of patients had metastatic BC. Among the BC patients, 62.2% were estrogen receptor-positive and HER2-negative with luminal-type BC, 24.3% had HER2-type BC, and 13.5% had TNBC. Details of patient characteristics are provided in Table 1.

**Table 1:** Characteristics of patients with breast cancer in the study.

Characteristics	No. (%)
**Healthy Volunteers**	54
**ALL patients**	185
**Age, years**	
Median	57
Range	27–89
**Stage**	
I	58 (31.3%)
II	61 (33.0%)
III	20 (10.8%)
IV	46 (24.9%)
**ER/PR status**	
ER-positive and/or PR-positive	144 (77.8%)
ER-negative and PR-negative	41 (22.2%)
**HER2 status**	
HER2 positive	45 (24.3%)
HER2 negative	140 (75.7%)
**Phenotype**	
Luminal	115 (62.2%)
HER2	45 (24.3%)
Triple negative	25 (13.5%)
**Grade**	
1	23 (12.4%)
2	92 (49.7%)
3	61 (33.0%)
unknown	9 (4.9%)
**Ki-67**	
Median	20
Range	1–91.6

### Expression pattern of cytokines in BC patients was distinct from healthy volunteers

**Figure 1:**
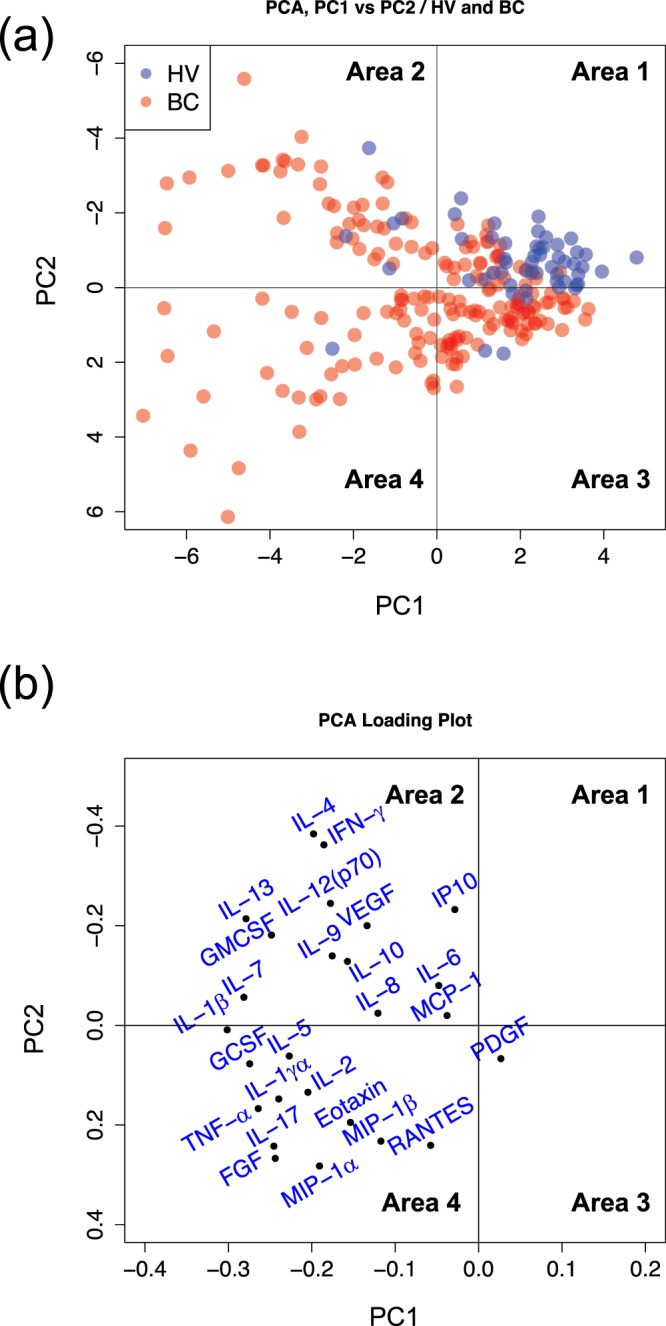
PCA score plot and corresponding loading plot for subjects. PCA score plot (**a**) and corresponding loading plot (**b**) of measured serum cytokines for healthy volunteers (blue) and BC patients (red). Area 1: PC1 > 0, PC2 < 0, Area 2: PC1 < 0, PC2 < 0, Area 3: PC1 > 0, PC2 > 0, Area 4: PC1 < 0, PC2 > 0.

PCA of 185 BC patients and 54 healthy volunteers showed that the former were clearly distinguishable from the latter (Fig. 1a). Almost all healthy volunteers were located in Area 1; there was only one healthy volunteer in Area 4. In contrast, BC patients were located in broader zones. A small overlap area between healthy volunteers and BC patients can be observed. Figure 1b shows the contribution of each cytokine to Primary Component 1 (PC1) and PC2. Cytokines, which existed in Area 2 (see Fig. 1b), contributed to Areas 1 and 2 (see Fig. 1a), such as in the form of IFN-γ and IL-13, and also existed in Area 4 (see Fig. 1b) and contributed to Areas 2 and 4 (see Fig. 1a), such as in the form of IL-17 and bFGF.

### Expression pattern of cytokines in cancer progression and phenotypes

**Figure 2:**
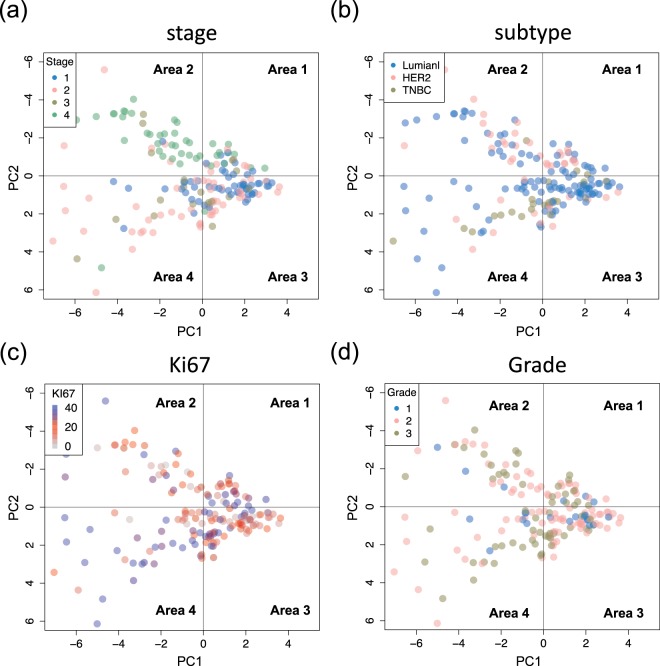
Correlation between PCA score and BC characteristics. PCA score plot with BC characteristics. (**a**) cancer stage, (**b**) cancer subtype, (**c**) % expression of Ki67 in the tumor, (**d**) grade (Elston-Ellis modification of Scarff-Bloom-Richardson grading system).

Next, we investigated the expression pattern in BC patients by subgroup plotting. Figure 2a depicts the PCA of the plot according to tumor staging. The patients with Stages 1, 2 and 3 (non-metastatic patients) were primarily located in Areas 3 and 4. In contrast, the patients with Stage 4 were primarily located in Areas 1 and 2. Figure 2b depicts the PCA of the plot of the BC phenotype. There was no signature for the luminal type, whereas the HER2 type tended to exist in the upper area (Areas 1 and 2) and the TNBC type tended to exist in the lower area (Areas 3 and 4). There was no significant pattern analyzed according to Ki67 and tumor grade (Fig. 2c,d).

### Correlation mapping of cytokines in BC patients and healthy volunteers

**Figure 3:**
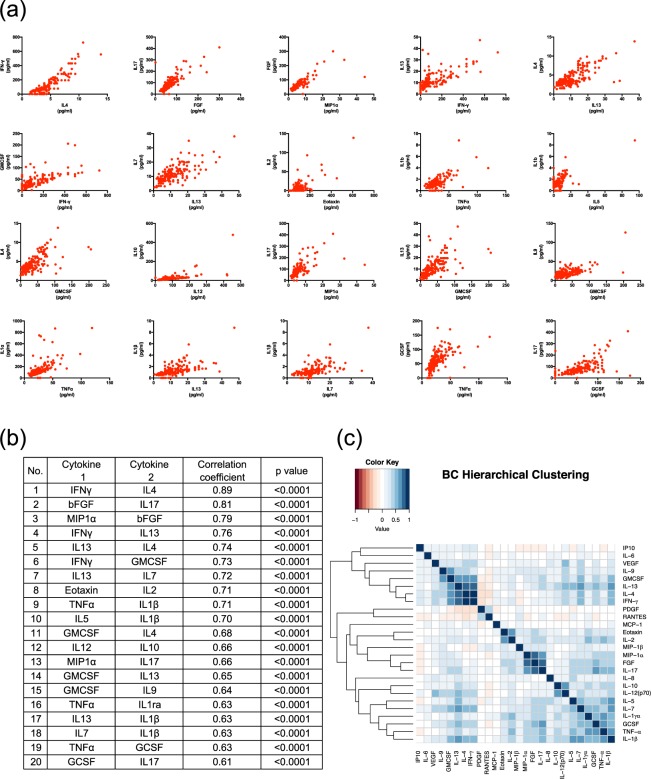
Correlation mapping of cytokines in BC patients and healthy volunteers. (**a**) Individual top 20 high correlation coefficient scatterplots. (**b**) The value of the correlation coefficient and the p-value of the top 20 high correlation coefficients. Statistical analysis was performed by the Pearson’s correlation test. (**c**) Heart map of cytokines’ network with cluster analysis in BC patients. Dark blue means high positive correlation and dark red mean high negative correlation.

Next, we investigated the correlation pattern of cytokine expression BC patients and healthy volunteers. Figures 3a,b show the top 20 strong correlation combinations of cytokines. Figure 3c shows the heat map of the cytokines’ network with cluster analysis in BC patients. In BC patients, there were three clusters: (1) VEGF, IL-9, GM-CSF, IL-13, IL-4, and IFNγ; (2) IL-8, IL-10, IL-12, IL-5, IL-7, IL-1α, GCSF, IL-1β, and TNFα; (3) IL-2, eotaxin, MIP1β, MIP1α, IL-17, and bFGF. These results indicate that there were specific correlation patterns in BC patients. We also checked the correlation between these cytokines and TGFβ, which has been established as essential for cancer progression, because of its prominent role in the regulation of cell growth, differentiation and migration^10^. Moreover, there was no significant correlation with another 26 cytokines (Supplementary Fig. S1).

### Prediction of BC prognosis and therapeutic effect by PCA and IL-17 expression pattern

**Figure 4:**
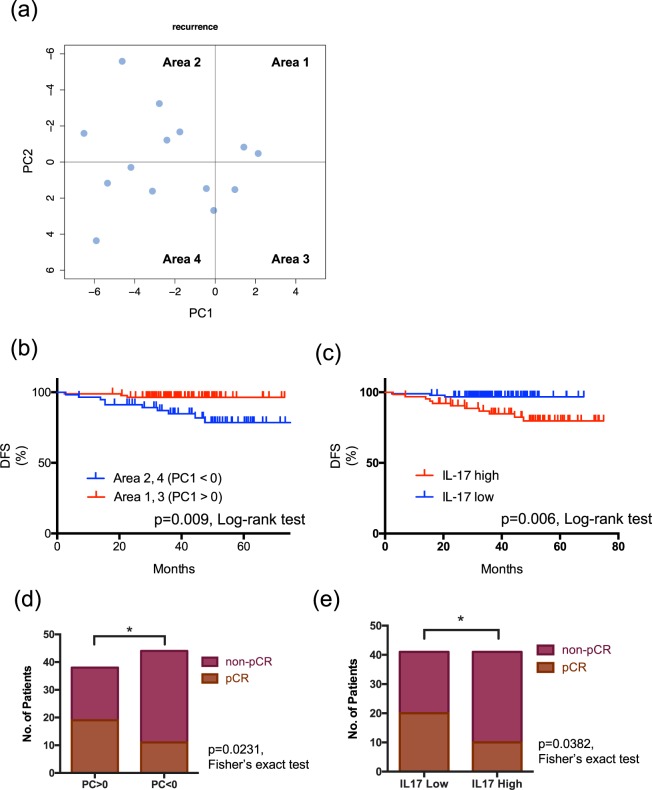
Prediction of BC prognosis and therapeutic effect by PCA and IL-17 expression pattern. (**a**) PCA score plots of the patients with Stage 1–3 operable BC. The blue circles plot patients in whom metastatic disease occurred, along with the score for PC1 (X-axis) and PC2 (Y-axis). (**b**,**c**) Kaplan-Meier estimate of overall survival in Stage 1–3 operable BC patients by PC value and IL-17 expression level. B: In the PC1 < 0 areas, there was significantly poor prognosis compared to patients who were in the PC1 > 0 area. Hazard ratio: 0.21; 95% CI, 0.075 to 0.681; P = 0.009 with the use of the log-rank test. (**c**) There was significantly poor prognosis in the high IL-17 group. Hazard ratio: 0.23; 95% CI, 0.078 to 0.662; P = 0.006 with the use of the log-rank test. (**d**,**e**) Correlation between PCA and IL-17 expression patterns and BC therapeutic effects among patients who received neoadjuvant chemotherapy. (**d**) A pCR was achieved in 36.6% of all cases (30/82), 25.0% of PC1 < 0 cases (11/44) and 50.0% of PC1 > 0 cases (19/38); p = 0.0231. (**e**) 24.4% of high IL-17 cases (10/41) and 48.8% of low IL-17 cases (20/41); p = 0.0382. Statically analysis was conducted using Fisher’s exact test.

We investigated the correlation between PCA expression patterns and BC prognosis. We followed up Stage 1–3 patients, with distant metastasis occurring in 14 out of 139 patients (Fig. 4a). Interestingly, these metastatic patients have distributed in the PC1 < 0 areas. Kaplan Mayer analysis showed that disease-free survival (DFS) was significantly shorter in patients in PC1 < 0 (Areas 2 and 4) compared to PC1 > 0 (Areas 1 and 3) (hazard ratio: 0.21; 95% confidence interval [CI], 0.075 to 0.681; P = 0.009 with the use of the log-rank test) (Fig. 4b). In subgroup analysis, the only grade was involved in DFS. Higher stage and HER2 and TNBC subtypes had also trend, but there was not significant due to small sample size (Supplementary Fig. S2). We also investigated the correlation between IL-17 and BC prognosis due to IL-17 being the strongest effector about PC1. There was significantly poor prognosis in the high IL-17 group, which was defined by median levels of IL-17 (hazard ratio: 0.23; 95% CI, 0.078 to 0.662; P = 0.006 with the use of the log-rank test) (Fig. 4c). Next, we investigated the correlation between PCA and IL-17 expression patterns and therapeutic effects among BC patients who received neoadjuvant chemotherapy (Tables 2 and 3). A pathological complete response (pCR) was achieved in 36.6% of all cases (30/82), 25.0% of PC1 < 0 cases (11/44), 50.0% of PC1 > 0 cases (19/38) (Fig. 4d), 24.4% of high IL-17 cases (10/41) and 48.8% of low IL-17 cases (20/41) (Fig. 4e). We checked alteration between cytokine profile at BC diagnosis and after neoadjuvant therapy using eight patient’s samples (Supplementary Fig. S3). Our findings indicate that there were significant alteration patterns in non-pCR with recurrence case. These results suggest that PCA based on serum cytokine levels could predict BC prognosis and therapeutic effects.

**Table 2:** Correlation between cytokine expression pattern and clinical features who received neoadjuvant chemotherapy (PCA).

Characteristic		PC1 < 0	PC1 > 0	p-value
		No. %	No. %	
**ALL patients**	82	44	38	
**Age, years**				0.929
Median	52.5	53	51.5	
Range	27–78			
**Subtype**				0.796
Luminal	29	17 (58.6%)	12 (41.4%)	
HER2	33	17 (51.5%)	16 (48.5%)	
Triple negative	20	10 (50.0%)	10 (50.0%)	
**Grade**				0.971
2	39	21 (53.8%)	18 (46.2%)	
3	43	23 (53.5%)	20 (46.5%)	
**Ki-67**				0.64
Median	30	30	30	
Range	10–91.6	10–91.6	10–91.6	
**Chemotherapy regimen**				
Anthracycline	45 (54.9%)	23 (51.1%)	22 (48.9%)	0.61
Taxane	79 (96.3%)	42 (53.2%)	37 (46.8%)	0.645
Platinum	27 (32.9%)	12 (44.4%)	15 (55.6%)	0.241
Trastuzumab	32 (39.0%)	16 (50.0%)	16 (50.0%)	0.595

**Table 3:** Correlation between cytokine expression pattern and clinical features who received neoadjuvant chemotherapy (IL-17).

Characteristic		IL-17 high	IL-17 low	p-value
		No. %	No. %	
**ALL patients**	82	41	41	
**Age, years**				0.273
Median	52.5	51	54	
Range	27–78	31–73	27–78	
**Subtype**				0.273
Luminal	29	16 (55.2%)	13 (44.8%)	
HER2	33	13 (39.4%)	20 (60.6%)	
Triple negative	20	12 (60.0%)	8 (40.0%)	
**Grade**				0.015*
2	39	25 (64.1%)	14 (35.9%)	
3	43	16 (37.2%)	27 (62.8%)	
**Ki-67**				0.79
Median	30	30	30	
Range	10–91.6	10–90	10–91.6	
**Chemotherapy regimen**				
Anthracycline	45 (54.9%)	24 (53.3%)	21 (46.7%)	0.292
Taxane	79 (96.3%)	46 (58.2%)	33 (41.8%)	0.771
Platinum	27 (32.9%)	15 (55.6%)	12 (44.4%)	0.701
Trastuzumab	32 (39.0%)	16 (50.0%)	16 (50.0%)	0.209

### Modeling of orthogonal projections to latent structures (OPLS) based on cytokine array provides information on patients with BC

Our data based on PCA and correlation analysis suggested the existence of three different types of cytokine patterns in the patients with BC. Based on the cytokine pattern, we performed OPLS-discriminant analysis (OPLS-DA) to predict the patients’ status and classification. In this analysis, healthy volunteers, patients with BC, operable patients with BC (Stages 1–3), metastatic patients with BC (Stage 4), TNBC and other types (luminal and HER2) are used as classification variants.

**Figure 5:**
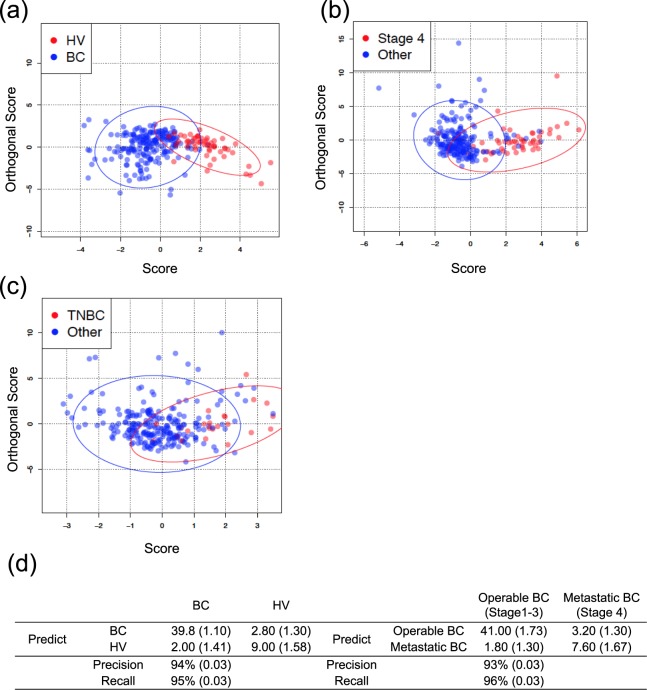
OPLS modeling based on cytokine array provides information on patients with BC. (**a–c**) OPLS-DA score plots of healthy volunteers and patients with BC, operable BC/metastatic BC, and TNBC/other types. The distributions of each sample are plotted by the red and blue circles, along with the score of the predictive component (X-axis) and the orthogonal component (Y-axis). The ellipse lines on the plots indicate the variance of the samples, which is drawn according to the Mahalanobis distance. (**d**) Summary of classification results (SD) calculated by k-fold cross-varidation (k = 5). Precision and recall refer to the relevance of the classification. See the main text for definitions.

As shown in Fig. 5a–c, we showed the scatterplot for each variant according to the trained model based on all sample data. The red and blue plot indicates each sample and the ellipse line represents the variance in each category. Intuitively, the smaller overlapped area of variance ellipses indicates the more precise prediction of classification. Our data show that the OPLS-DA model could distinguish BC patients from healthy volunteers (Fig. 5a). In the analysis on operable and metastatic BC, even the overlap of variance ellipses is slightly larger than in the previous plot, while the data also suggest that our OPLS-DA model may predict both operable and metastatic BC (Fig. 5b).

In contrast, in the analysis on TNBC and other types, a large part of each ellipse line overlap, indicating that our OPLDS-DA model makes it difficult to distinguish TNBC from other types (Fig. 5c). To validate our OPLS-DA models, we performed k-fold cross-validation and predicted classification performance, here we used k = 5. The analysis of TNBC and other types could not be performed because of the insufficient sample size. Precision was calculated by using the following formula: precision = (true positive)/(true positive + false positive). The recall was also calculated using the following formula: recall = (true positive)/(true positive + false negative). As shown in Fig. 5d, the OPLDS-DA model for predicting BC patients and healthy volunteers shows satisfactory precision and recall values.

Moreover, the OPLDS-DA model for predicting both operable and metastatic BC shows potent precision and recall values. These results indicate that OPLS-DA analysis based on serum cytokine levels can precisely predict patients with BC. More importantly, our data showed that OPLS-DA analysis based on serum cytokine levels could predict operable or metastatic BC with a 90% probability.

### Correlation between serum cytokine expression and immune profile on BC tissues

**Figure 6:**
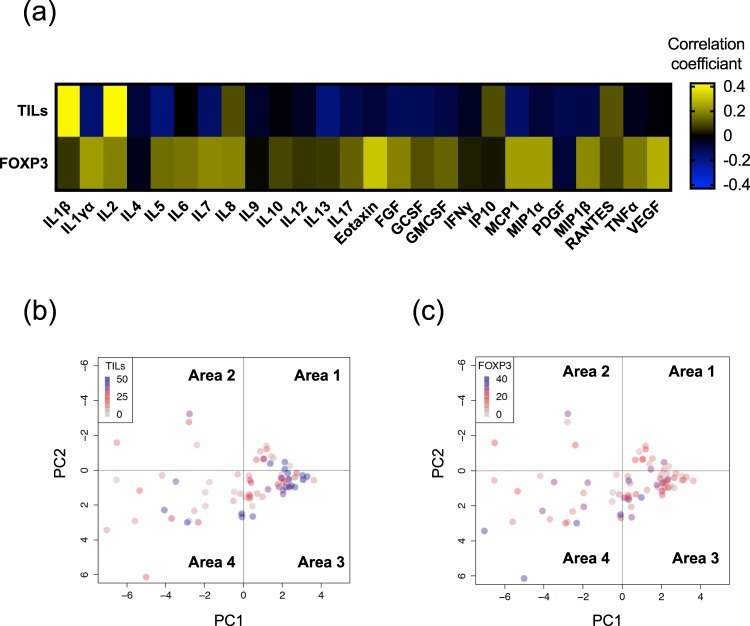
Correlation between serum cytokine expression and immune profile on BC tissues. (**a**) The value of the correlation coefficient between serum cytokine expressions and immune profile on BC tissues. The immune profile was evaluated TILs and FOXP3 on FFPE samples. Statistical analysis was performed by the Pearson’s correlation test. (**b**,**c**) PCA score plots of the patients with Stage 1–3 operable BC. (**b**) TILs (% stromal area), (**c**) FOXP3 (% TILs).

The previous study revealed that immune profile, such as tumor infiltrating lymphocytes (TILs) and FOXP3, on BC tissues has both predictive and prognostic values in BC^11,12^. However, the correlation between serum cytokine expression and immune profile in BC tissues is still unclear. We analyzed this correlation by evaluating TILs and FOXP3 in breast cancer tissues to uncover the immune response in breast cancer tumor microenvironment. We evaluate 83 paired samples in this study and characteristic of patients is in Supplementary Table S1. Interestingly, the majority of cytokines have a negative correlation with TILs and have a positive correlation with FOXP3 (Fig. 6a–c). These results suggest that high TILs correlates with low pro-inflammatory cytokine expression status and high FOXP3 correlates with high pro-inflammatory cytokine expression status in BC.

## Discussion

Tumor tissue is the gold standard for clinical and investigational sequencing. However, major barriers exist regarding acquisition and utility, such as inconvenience from a scheduling perspective, the cost of patient care, and invasive procedures for patients. The primary limitation of tissue biopsy is heterogeneity, which characterizes most advanced cancers^13^, with heterogeneity existing between metastases within the same patient. To overcome the limitations of tissue biopsy, less invasive techniques capable of capturing tumor heterogeneity and the molecular changes that cancer cells undergo when they are exposed to therapy are needed^14^. The blood test, which is the appropriate and most straightforward measure for mass screening, is widely used in the testing of several diseases including metabolic syndrome.

In this study, we presented a novel approach of cytokine networks based on the comprehensive analysis of 26 kinds of expression inflammatory cytokine expression patterns, followed by chemometric analysis. We revealed the specific cytokine expression patterns between patients with BC and healthy volunteers and the specific cytokine signature in metastatic patients with BC.

Recent studies have suggested the existence of complex crosstalk between the tumor and host, which plays a crucial role in tumor initiation, growth, metastasis, and drug resistance. The inflammatory cytokine is the main component of this crosstalk. For instance, several inflammatory cytokines are crucial in the pre-metastatic niche in several animal models. There were several reports, which described cytokines and BC progression. Wang *et al*. reported that serum IL-6, IL-8, IL-10, SCC-Ag, and CYFRA 21-1 may serve as potential markers for predicting metastasis and prognosis of BC^15^. Circulating tumor cells (CTCs), which is increased with BC progression and metastasis, involved in cytokine expression in BC. Vilsmaier *et al*. reported that IL-1α is a candidate marker for the release of tumor cells and IL-1β, IL-12, sFlt1 and PlGF appears to be related to CTCs release in patients with BC^16,17^. Another group showed that pro-inflammatory cytokines TNFα, IL-6, CSF-1, and IFNγ correlated with disseminated tumor cells in BC^18^. We demonstrate that cytokine expression pattern correlated with both immune profiles on BC tissues (Fig. 6a). Inflammation in a tumor microenvironment comprises infiltrating immune cells that secrete cytokines, chemokines, and growth factors to which the tumor responds^1,19^. Previously, we revealed that BC progression alters immune profile in peripheral blood mononuclear cells (PBMCs)^9^. FOXP3 expression in PBMCs is one of the genes correlated with BC progression, and we discovered that FOXP3 in BC tissues also is correlated with serum cytokine expression pattern. Overall, our results may uncover significant immune response in BC tumor microenvironment.

Consistent with these findings, clinical studies have shown the upregulation of several inflammation cytokines including G-CSF, IL-6, and IL-17 in patients with BC^5^. Meanwhile, recent reports have shown that IL-17-producing cells increased in tumor tissues and peripheral blood from different cancer patients^20^ ^{Numasaki, 2004 #707^. In contrast, a blood test to measure IL-17 levels is the more feasible way to evaluate the significance of IL-17 in tumor microenvironments, compared with measuring IL-17-producing cells. In non-small-cell lung cancer and hepatocellular carcinoma, serum IL-17 levels were correlated with poor prognosis^21,22^. Here, we are the first to show that serum IL-17 is up-regulated in BC patients and that high levels of IL-17 are also correlated with poor clinical outcomes.

Recently, preclinical models have shown that IL-17 mediates cancer progress via various mechanisms^23–28^. Coffelt *et al*. reported that increasing systemic IL-17 levels leads to the up-regulation of G-CSF, which subsequently causes neutrophil expansion and alteration of the neutrophil phenotype. Another study reported the effects of IL-17 and bFGF on mesenchymal stem cell (MSC) growth and elucidated the signaling pathways involved. These results indicated that IL-17 increased the frequency of colony-forming unit fibroblast (CFU-F), as well as MSC proliferation in a dose-dependent manner, while bFGF supplementation induced an increase in cell proliferation^27^. Consistent with these findings, our data indicated that the interaction of serum IL-17 levels with an increased level of both bFGF and G-CSF are correlated with poor DFS among BC patients. While IL-17- and IL-17-producing cells are significantly insensitive towards chemotherapy in experimental settings^29^, little is known about the correlation between systemic IL-17 levels and sensitivity towards chemotherapy in clinical settings, especially concerning BC. Although there are limitations to our study, given that patient characteristics include all subtypes and involve various chemotherapy regimens, our data indicate that systemic IL-17 levels are correlated with pathological complete response rates in BC patients. To overcome such limitations, we need to further investigate the correlation between serum IL-17 levels and clinical outcomes in larger-volume clinical trials.

We acknowledge that this study has several other limitations. Notably, this study is retrospective, meaning that it is difficult to evaluate correlations between cytokine diversity and clinical outcome due to treatment and phenotype bias. We should validate using individual cohort to enhance our conclusion. To overcome this limitation, we are planning to validate using two ongoing clinical trial data set. The first one is to investigate the efficacy and safety of dual-HER2 blockage therapy in HER2-positive breast cancer (Clinical trial information: UMIN000007576). The other one is to evaluate the safety, antitumor effect and improved prognosis of Nivolumab with radiation therapy in patients with HER-2-negative metastatic breast cancer have bone metastasis that possible to irradiate (Clinical trial information: UMIN000026046). These clinical trials will cover all phenotypes. Additionally, we will investigate not only cytokine expression pattern but also tumor immune microenvironment status using CyTOF analysis and RNA-seq analysis of PBMCs. Then, we will confirm the correlation between cytokine expression pattern and tumor microenvironment status.

In this study, at least in the evaluations of BC development and progression, our analysis could be potentially relevant as we revealed them by OPLS-DA (Fig. 5). To further strengthen our model, we performed k-cross-validation as it is used for the development of biomarker classifiers from high dimensional data set^30^. Interestingly, results of k-fold cross-validation showed high accuracy to distinguish BC from HV and stage IV patients from stage I-III patients. Additionally, the standard deviation value is less than 5% both recall and precision rate. Therefore, we think the risk of over-learning is very low. Considering the application, our data are crucial because our results suggest that serum analysis is a useful approach to provide information, for example, on the subtype of cancer, inflammation status and prognosis by a 12.5-µl serum sample. Regarding accessibility, for example, in terms of low invasion, established method and low cost, the comprehensive analysis of inflammatory cytokines in serum could be useful for monitoring inflammation status in the use of antagonizing/neutralizing antibodies to address inflammatory cytokine signaling.

## Methods

### Serum samples

Serum from healthy volunteers, early-stage BC patients, and metastatic BC patients was collected by the Department of Breast Surgery, Kyoto University Hospital, and the Resource Center for Health Science, Kyoto, Japan. All the BC patient’s samples were collected at the BC diagnosis between April 2011 and March 2015. Eight patients had two-point samples, both at diagnosis and after neoadjuvant therapy. Written informed consent was given by all patients before collection. All study protocols were approved by the Ethics Committee for Clinical Research, Kyoto University Hospital (Authorization Number G424) and performed according to the Declaration of Helsinki and the Ethical Guidelines for Epidemiological Research of the Ministry of Education, Culture, Sports, Science, and Technology and the Ministry of Health, Labour, and Welfare of Japan. Samples were frozen in liquid nitrogen and stored at −80 °C within 2 hours from sample collection. We checked if sample storage period effects on cytokine expression in this study (Supplementary Fig. S4). All of the value of correlation coefficient were within 0.2, and there was no significant distribution in the characteristic of patients.

### Cytokine and chemokine measurement

The cytokines in serum samples, IL-1β, IL-1γα, IL-2, IL-4, IL-5, IL-6, IL-7, IL-8, IL-9, IL-10, IL-12 (p70), IL-13, IL-15, IL-17, granulocyte-macrophage-colony-stimulating factor, granulocyte colony-stimulating factor, interferon gamma (IFN-γ), monocyte chemoattractant protein-1, macrophage inflammatory protein-1α (MIP-1α), macrophage inflammatory protein-1β (MIP-1β), PDGF-BB, RANTES, VEGF, bFGF and TNF-α were measured with a Bio-Plex multiplex assay system (Bio-Rad, Hercules, CA) according to the manufacturer’s instructions. This analysis was conducted on 12.5-µl serum sample from each patient.

### Analysis of the immune profile on BC tissues

The paraffin sections were rehydrated and immersed in 3% hydrogen peroxide for 10 min to quench endogenous peroxidase activity. Epitope retrieval was performed by heating the sections at 95 °C in Target Retrieval Solution (pH 9, S2375; Dako) for 30 min in an electric pressure cooker (SR-P37; Panasonic, Tokyo, Japan). All incubations after the blocking step were performed at room temperature. The wash buffer was 0.05 M Tris-buffered saline with 0.05% Tween 20 (pH 7.6). Following blocking with 5% normal goat serum (Abcam) in PBS for 10 min, the sections were incubated with anti-FOXP3 antibodies (236 A/E7; Abcam) for 1 h.

Histopathologic assessment of the percentage of TILs and FOXP3 was performed on one representative immunohistochemical section of a tumor using methods recommended by the International TILs Working Group 2014^18^. % TILs was defined as the percentage of lymphocyte area per tumor area *in vivo* samples. Areas of *in situ* carcinomas, normal lobules, necrosis, hyalinization, and crush artifacts were not included. Histopathologic evaluation of TILs was performed by two breast pathologists who scored each case independently in a blind manner. The mean values of both observers were obtained as the final scores for each case.

### Statistics and data analysis

Multivariate statistical analysis (PCA and OPLS-DA) was performed in order to evaluate the measured cytokine data using R version 3.3.1 (R Foundation for Statistical Computing, 2016) and the R ropls package version 1.8.0. PCA can project higher-dimension data into lower-dimensional space in order to visualize the distribution of complex data. Typically two-dimension is used as the lower-dimensional space. Usually, PCA results are represented by a score plot and a loading plot. In a score plot, samples expressing similar cytokine values are plotted in the near position, while the spatial groups of similar samples are recognized as a pattern. In a loading plot, the positions of each point corresponding to the cytokine variables describe the correlation with the PCs, such as PC1 and PC2. The loading plot implies which cytokines mainly contribute to forming the pattern in the score plot. In our PCA calculation, a few outlier samples, which were more than 10–100 times larger than the average of the distribution, were omitted because substantial outlier values collapse the pattern. OPLS-DA is a potent statistical tool, which provides insights into distinguishing between groups of high-dimensional spectral data^31^. The score axis is the predictive component, which is the variation correlated to the factor of interest. The orthogonal score axis is the first component of the uncorrelated variations. A χ^2^ test (two-sided, α = 0.05) was used to compare the populations between groups in the score plot, using SPSS 17.0 (Chicago, IL, USA).

k-fold-cross-validation: The dataset was randomly partitioned into k = 5 disjoint subsets to be used as a training and testing sets to validate the Delphi exercise-approved diagnostic criteria. Four subsets were used as the training set to build a diagnostic model, and the remaining subset was used as the testing set to test the model. This process is repeated five times for each dataset, each round with a different training set and complementary testing set. Model performance was estimated by calculating precision and recall which definitions were described at the results section. The averages and standard deviations over the 5 datasets were calculated.

## Supplementary information

Supplementary information

## Data Availability

The datasets generated during and analyzed during the current study are not publicly available due to protecting participant confidentiality but are available from the corresponding author on reasonable request.
