# Supplementary material for: Alteration of specific cytokine expression patterns in patients with breast cancer
Source: Sci Rep. 2019 Feb 27;9:2924. doi: 10.1038/s41598-019-39476-9 (PMC6393524; doi:10.1038/s41598-019-39476-9)
Supplement: Supplementary file 1 — Supplementary information [file 41598_2019_39476_MOESM1_ESM.pdf]

Alteration of specific cytokine expression patterns in patients with breast cancer

Kosuke Kawaguchi[1]†*, Masashi Sakurai[2]†, Yasuko Yamamoto[3], Eiji Suzuki1, Moe Tsuda[1], Tatsuki R. Kataoka[4], Masahiro Hirata[4], Mariko Nishie[1], Takashi Nojiri[5], Motofumi Kumazoe[5] Kuniaki Saito[2, 3], Masakazu Toi[1]

1Department of Breast Surgery, Kyoto University Graduate School of Medicine, Kyoto, Japan
2 Human Health Sciences, Graduate School of Medicine and Faculty of Medicine, Kyoto University, Kyoto, Japan
3 Department of Disease Control and Prevention, Fujita Health University Graduate School of Health Sciences, Toyoake, Aichi, Japan
4Department of Diagnostic Pathology, Kyoto University Hospital, Kyoto, Japan
5Department of Biochemistry, National Cerebral and Cardiovascular Center Research Institute, Suita-City, Osaka, Japan
†Contributed equally

*Address for reprint requests and correspondence: Kosuke Kawaguchi
Department of Breast Surgery, Kyoto University Graduate School of Medicine,
54 Shogoin-kawaharacho, Sakyo-ku, Kyoto 606-8507, Japan
Telephone: +81-75-751-3660
Fax: +81-75-751-3616
Email address: kkosuke@kuhp.kyoto-u.ac.jp

Supplementary Figure S1

A

	IL1β	IL1γα	IL2	IL4	IL5	IL6	IL7	IL8	IL9	IL10	IL12	IL13	IL17
r	0.06	0.10	0.07	-0.01	-0.03	0.06	-0.03	-0.03	0.00	0.01	0.05	-0.02	-0.09
R squared	0.00	0.01	0.01	0.00	0.00	0.00	0.00	0.00	0.00	0.00	0.00	0.00	0.01
P value	0.76	0.62	0.72	0.97	0.88	0.76	0.90	0.90	0.98	0.96	0.79	0.91	0.67
	Eotaxin	FGF	GCSF	GMCSF	IFNγ	IP10	MCP1	MIP1α	PDGF	MIP1β	RANTES	TNFα	VEGF
r	0.07	0.05	0.07	0.06	0.07	0.17	-0.02	0.03	-0.29	-0.07	-0.10	0.07	0.06
R squared	0.00	0.00	0.01	0.00	0.00	0.03	0.00	0.00	0.08	0.00	0.01	0.00	0.00
P value	0.74	0.82	0.72	0.75	0.73	0.38	0.93	0.88	0.14	0.74	0.62	0.73	0.78

B

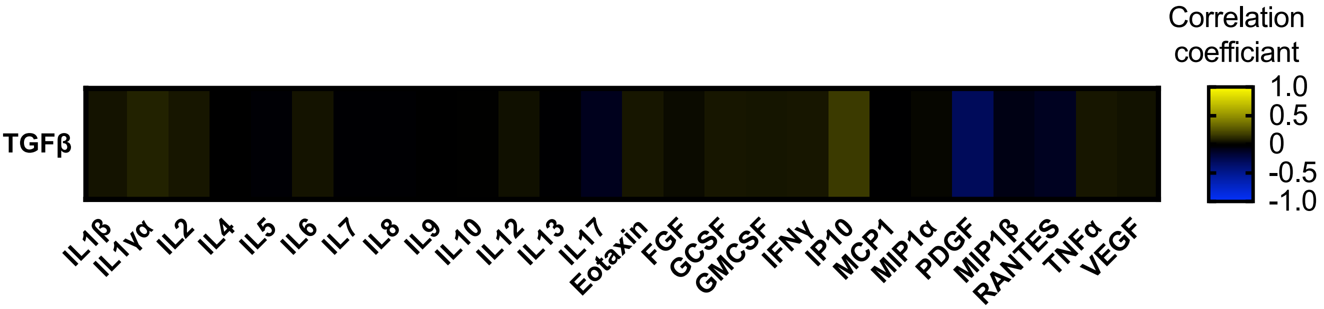

C

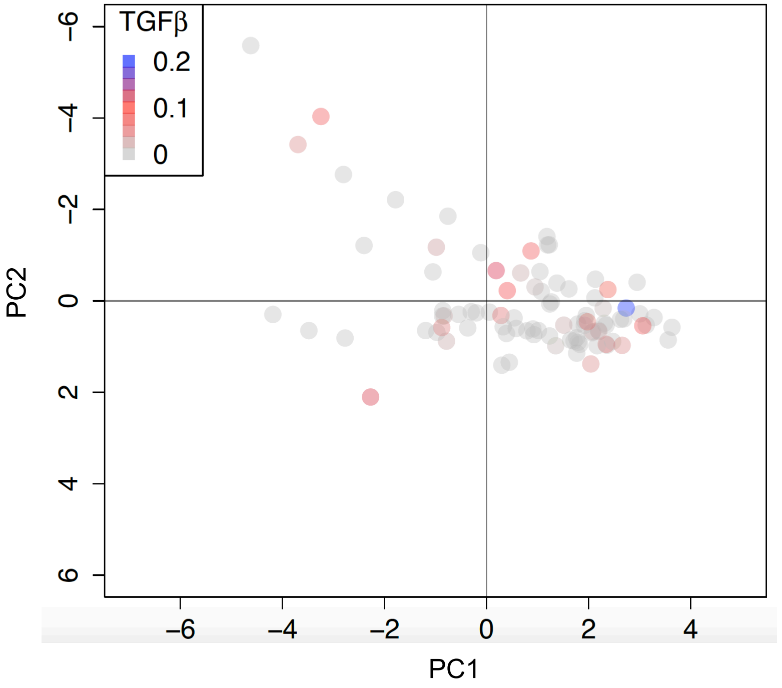

Supplementary Figure S2

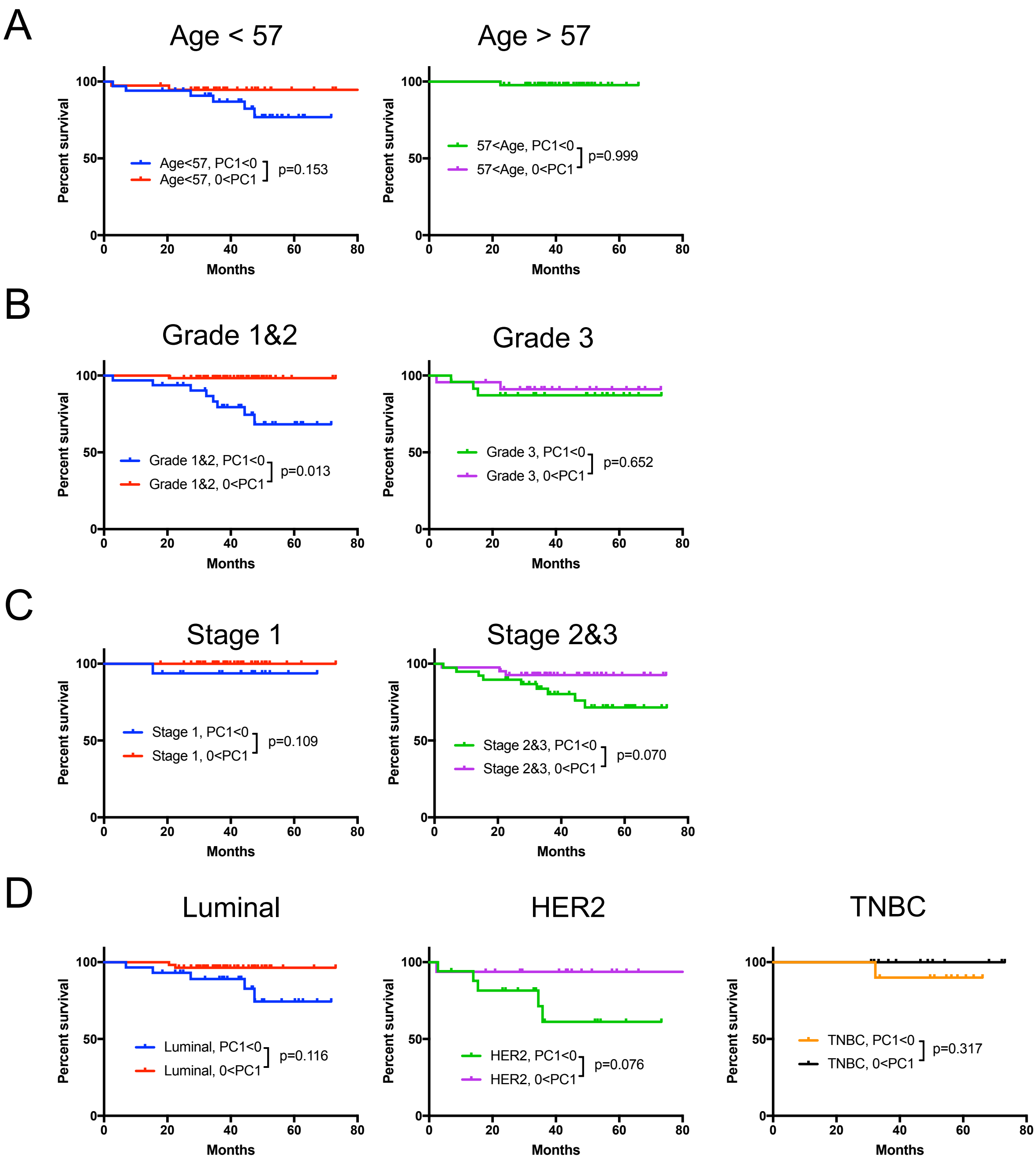

Supplementary Figure S3

A

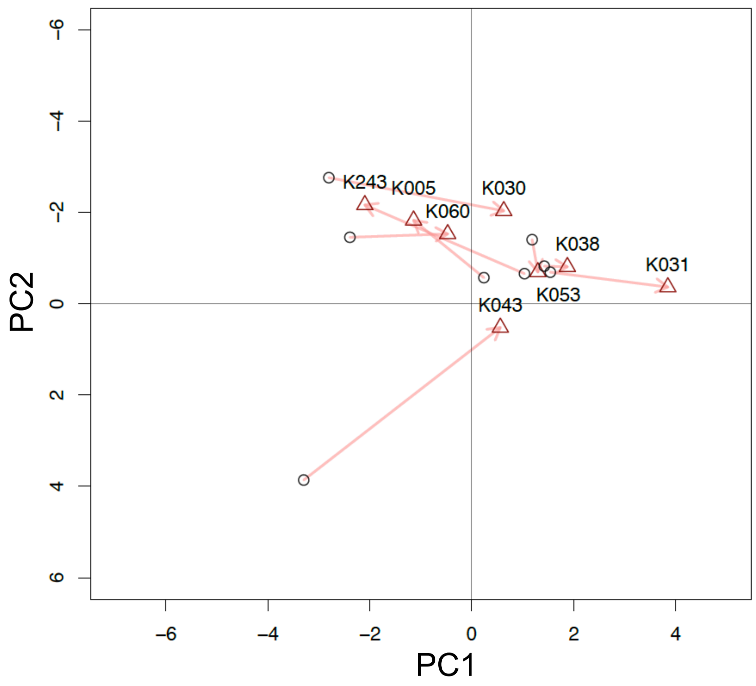

B

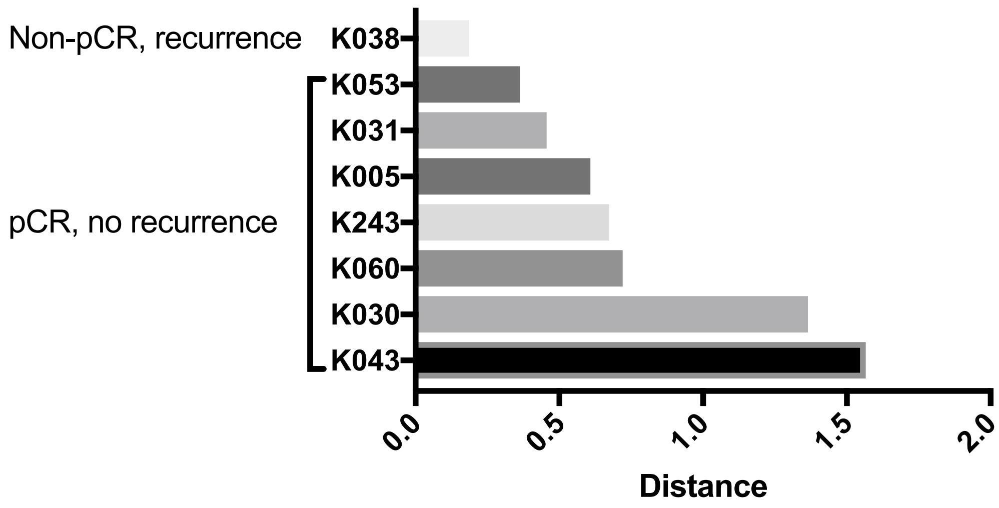

Supplementary Figure S4

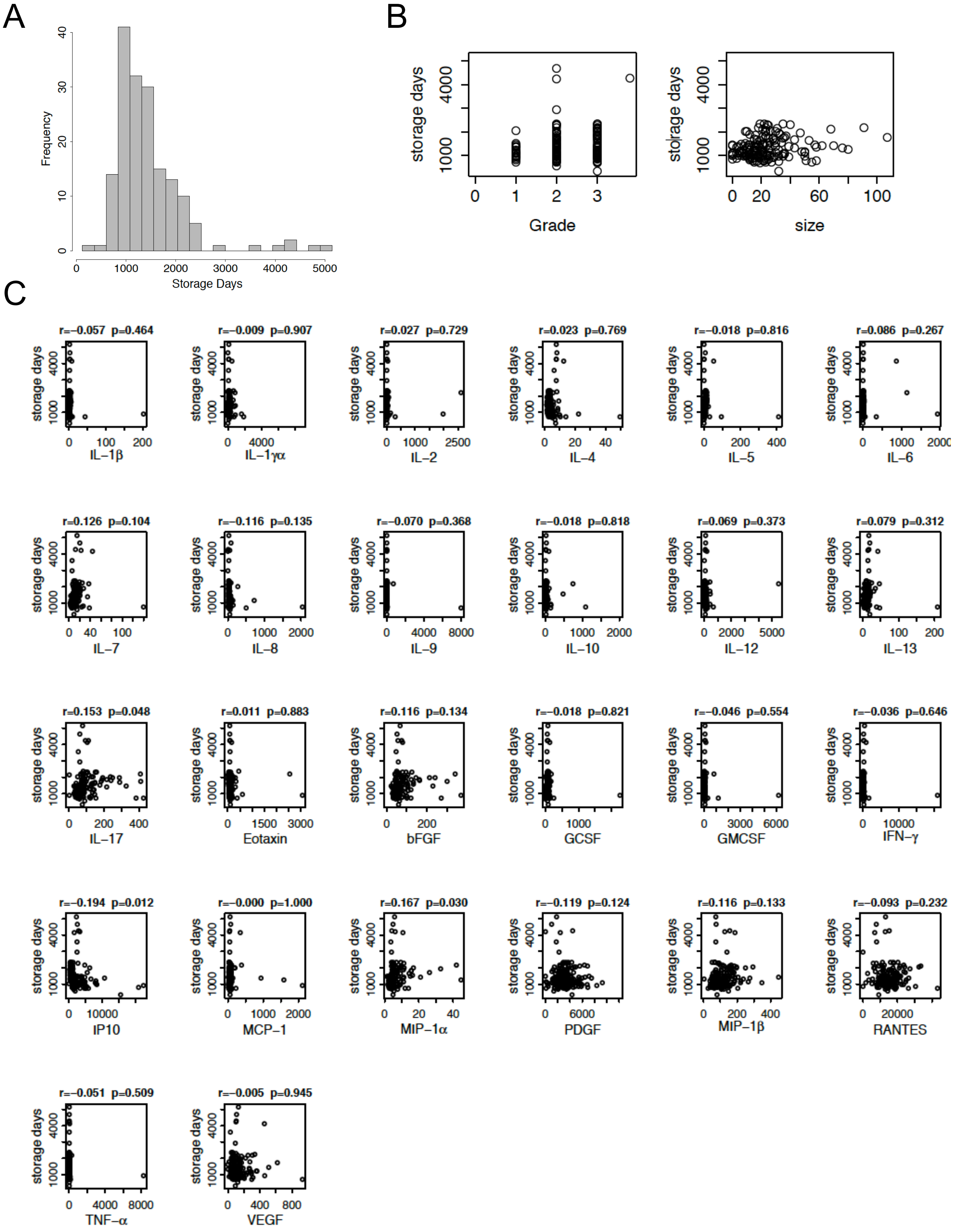

Supplementary Table S1

A

Characteristics of patients with immune profile

Characteristeic	No.	(%)
**ALL patients**	83	
**Age, years**		
Median	54.5	
Range	30-89	
**Stage**		
I	35	(42.2%)
II	36	(43.4%)
III	11	(13.3%)
Ⅳ	1	(1.2%)
**ER/PR status**		
ER-positive and/or PR-positive	60	(72.3%)
ER-negative and PR-negative	23	(27.7%)
**HER2 status**		
HER2 positive	22	(26.5%)
HER2 negative	61	(73.5%)
**Phenotype**		
Luminal	45	(54.2%)
HER2	22	(26.5%)
Triple negative	16	(19.3%)
**Grade**		
1	10	(12.0%)
2	41	(49.4%)
3	30	(36.1%)
unknown	2	(2.4%)
**Ki-67**		
Median	22	
Range	1-91.6	
